# Adjuvant chemotherapy for resected non-small-cell lung cancer: future perspectives for clinical research

**DOI:** 10.1186/1756-9966-30-115

**Published:** 2011-12-29

**Authors:** Maria Bonomi, Sara Pilotto, Michele Milella, Francesco Massari, Sara Cingarlini, Matteo Brunelli, Marco Chilosi, Giampaolo Tortora, Emilio Bria

**Affiliations:** 1 Medical Oncology, Azienda Ospedaliera Universitaria Integrata (AOUI), Verona, Italy; 2 Department of Medical Oncology, Regina Elena National Cancer Institute, Roma, Italy; 3 Pathology, AOUI, Verona, Italy

**Keywords:** adjuvant chemotherapy, early stage lung cancer, stage Ib, prognostic predictive classifiers

## Abstract

Adjuvant chemotherapy for non-small-cell lung carcinoma (NSCLC) is a debated issue in clinical oncology. Although it is considered a standard for resected stage II-IIIA patients according to the available guidelines, many questions are still open. Among them, it should be acknowledged that the treatment for stage IB disease has shown so far a limited (if sizable) efficacy, the role of modern radiotherapies requires to be evaluated in large prospective randomized trials and the relative impact of age and comorbidities should be weighted to assess the reliability of the trials' evidences in the context of the everyday-practice. In addition, a conclusive evidence of the best partner for cisplatin is currently awaited as well as a deeper investigation of the fading effect of chemotherapy over time. The limited survival benefit since first studies were published and the lack of reliable prognostic and predictive factors beyond pathological stage, strongly call for the identification of bio-molecular markers and classifiers to identify which patients should be treated and which drugs should be used. Given the disappointing results of targeted therapy in this setting have obscured the initial promising perspectives, a biomarker-selection approach may represent the basis of future trials exploring adjuvant treatment for resected NSCLC.

## Background

Adjuvant chemotherapy (ACT) for NSCLC still represents a major topic in clinical oncology. According to guidelines from the European Society of Medical Oncology (ESMO) [1], American Society of Clinical Oncology (ASCO) [2], National Comprehensive Cancer Network (NCCN) [3] and American College of Chest Physicians (ACCP) [4,5] cisplatinum based ACT is now considered a standard treatment for resected stage II-IIIA with an estimated survival benefit of 4-5% at 5 years. Nevertheless, many issues such as the management of stage IB, the role of postoperative radiotherapy (PORT), the treatment of elderly-unfit patients, the best regimen and schedule to be used and the long term effects of ACT, are still under investigation. Moreover, the narrow ACT therapeutic index (i.e. limited survival benefit with considerable toxicity) requires a careful assessment of expected risks and benefits for each patient.

To date, no other prognostic or predictive factors beyond pathological stage have been prospectively validated. Molecular markers or classifiers could better identify which patients should be treated with, or spared by, chemotherapy and which drugs should be better used (assuming a differential sensitivity to a particular agent/regimen). Despite researchers' efforts, this still represents an unmet medical need. The purpose of this review is to summarize the available evidences on ACT in the context of the new recent advances in the field of translational and bio-molecular research.

## The historical perspective: so far, so good?

Since the NSCLC Collaborative Group landmark meta-analysis, which first indicated a small benefit in favor of ACT for resected NSCLC [6], many randomized clinical trials have been released with conflicting results.

The Adjuvant Navelbine International trial association (ANITA) trial [7] and the National Cancer Institute of Canada Clinical Trial Group (NCIC CTG) JBR-10 trial [8] confirmed the OS benefit of Cisplatinum and Vinorelbine adjuvant chemotherapy. The former enrolled stage I-IIIA patients and allowed the use of PORT, while the latter was limited to IB-II without radiotherapy. The OS improvement was 8.6% and 15% at 5 years, with HR of 0.79 and 0.7 respectively, maintained at longer follow up [7,9]. The International adjuvant lung cancer trial (IALT) [10], despite positive results at first analysis (4% reduction in the risk of death in enrolled stage II-IIIA patients undergoing platinum based ACT with either etoposide or vinca alkaloids [11]), failed to maintain the same benefit with longer follow up. So did the "stage IB-focused" CALBG 9633, which used a carboplatinum based regimen [12,13]. The negative results of the Big Lung Trial (BLT) [14], the Adjuvant Lung Project Italy (ALPI) [15] and ECOG 3590 [16] further jeopardized evidence on ACT.

The description of each trial is beyond our aim, however differences in study design, patient selection, schedule/regimen administered, and use of PORT could partially explain the conflicting outcomes [17].

In 2008 the LACE meta-analysis pooled individual patients' data from 5 of these trials [7,8,10,14,15] (using modern platinum based -ACT and conducted after 1995; 4584 patients) and showed a statistically significant absolute OS benefit of 5.4% (HR for death = 0.89; 95% CI 0.82-0.96; p = .005) [18].

The results of other meta-analysis [19-22] showed similar HR/RR for death for platinum based -ACT (0.86 -0.93), regardless of the method used (individual patients' data vs abstracted data). So did the recent update of the NSCLC-meta-analysis Collaborative Group (HR 0.89. 95% CI 0.82-0.97, p = 0.006 HR 0.86. 95% CI 0.81-0.92, p < .0001, absolute OS benefit: 4% at 5 years for the overall population)[23]. In a larger setting, community based surveys or multinstitutional database analyses show an increasing employment of ACT (with a consequent survival improvement) [24-29]. These data, interpreted with the caution requested by their retrospective and not randomized fashion, suggest that the benefit may also be extended into the context of patients treated in routine clinical practice.

With the aim to better interpret the quantitative and qualitative differences among randomized clinical trials results, IALT, JBR-10 and ANITA were analyzed with a bayesian approach, weighting the results on the basis of continuously updated outcome hypotheses [30]. Nevertheless, the 13% relative death risk reduction corresponding to an absolute 4-5% survival benefit did not increase overtime when considering the former NSCLC Collaborative Group meta-analysis publication [6] and its recent update [23]. These small benefit strongly call for an optimization of the therapeutic index of adjuvant treatment.

## The stage IB dilemma: Does (just) the size matters?

The management of stage IB (according to the 6th TNM edition) is still controversial. To date, evidence show that benefit from adjuvant chemotherapy for stage IB, if any, is small: 43 IB patients should be treated for one to benefit (number needed to treat, NNT), nearly 3 times the 15 NNT for stage II-IIA [2]. In addition, available results come from a trial with limited sample size (CALGB 9633) and from subgroup analysis of other randomized trials (with few enrolled stage IB patients), both underpowered to detect the small differences expected in OS.

In this regard, both the CALBG 9633, specifically designed for stage IB disease, and subgroup analyses of the IALT, JBR-10 and ANITA [7,8,11] trials failed to demonstrate any survival benefit [13]. A possible beneficial effect was seen for tumors larger than 4 cm (in comparison with smaller tumors) in CALBG 9633 (HR 0.69; p = .043 vs HR = 1.12; p = .32) [13] and JBR-10 (HR 0.66 vs 1.73) [8]. Since both these analyses were post-hoc, results are not conclusive, given also that the benefit lowers overtime [31]. Similarly, in LACE meta-analysis stage IB only trended toward an OS benefit. The HR was 0.93 (95% CI 0.78-1.10), against 0.83 and 1.14 for stage II-III and IA, respectively [18].

The subgroup analysis from the NSCLC CG meta-analysis update according to stage [23] and limited to platinum-based regimens, showed an identical 5 years OS improvement of 5% for stage IB (from 55 to 60%), stage II (from 40 to 45%) and stage III (from 30 to 35%), with a non significant test for trend (p = 0.13) [23].

A 11% 5 years survival gain (from 74% to 85%) was observed in Japanese patients with resected stage Ib adenocarcinoma treated for 2 years with oral Uracil-Tegafur. Although promising, these results cannot be directly extendend to Western countries whereas Uracil-Tegafur has not been reliably tested so far [32].

Conducting prospective trials restricted (and powered) for stage IB patients would be the only way to unravel this issue. However, the prohibitively large sample size required undermines the feasibility of such an approach [33].

In addition, other (molecular) prognostic factors are needed to identify among these borderline patients, those at higher risk. Nonetheless, the worse prognosis observed with increasing T size has been recognized in the VII TNM edition. T2 was divided into T2a (3-5 cm) and T2b (5 -7 cm), with a OS of 58 and 49% at 5 years, respectively (p < .0001) [34]; T2bN0 was upstaged to stage IIA [35]. Correlation with the new staging system failed to validate the 5 cm cut-off in the 9-years update of CALGB 9633, showing a trend towards a significant benefit for adjuvant treatment for patients with tumors > 7 cm [HR = 0.53; p = .051] [31], although interaction should be investigated.

Recent studies investigated further pathological prognostic factors for resected VII edition-stage IB (T2aN0), such as the presence of microscopic vascular invasion [36] or intratumoral vascular and/or visceral pleural invasion [37]. Although promising, these results require a prospective external validation.

Finally, the question of 'which stage IB deserves adjuvant treatment' remains still unanswered. Size may represent a selection criterion, while awaiting for more powerful pathological and biological predictors.

## Post Operative Radiotherapy (PORT): has the 1998 sentence expired?

Few and underpowered randomized clinical trials exploring the role of PORT in patients after resection of NSCLC have been conducted from the early 90s, with inconclusive results. In order to look for a small survival benefit, the individual patients' data PORT meta-analysis (initially including 9 randomized clinical trials) was performed [38]. The last update (11 trials, 2343 patients) showed a statistically significant detrimental effect on OS for patients receiving PORT (HR = 1.18; 95% CI 1.07-1.31; p = .0001; 5% 2-years absolute difference). Similar conclusions were reached for local and distant Recurrence-Free Survival (RFS) (HR = 1.12, p = .03 and HR = 1.13, p = .02, respectively). A highly significant interaction according to stage and nodal status was detected, indicating a substantial absence of PORT effect in stage III or N2 patients (HR 0.99 and 0.97), restricting the detrimental difference to lower stage disease [39].

Abandoned techniques, such as Cobalt-60, large irradiation fields (including the entire mediastinum), different total doses (30-60 Gy), unconventional daily fractions (up to 2,6-3 Gy) represent some of the limitations of the trials included in the PORT meta-analysis, thus undermining its validity in a modern setting. One of the hypotheses is that the excess in non cancer related deaths, mainly due to cardiovascular and lung toxicity, could have outweighed the potential small benefit on local recurrence [40].

The effect of PORT was also assessed in an unplanned analysis of the ANITA trial. Although no formal statistical comparison could be made between subgroups, a positive effect of PORT was suggested for N1 patients in the control arm and for N2 patients overall [41]. The latter derived the largest benefit from the association of adjuvant chemotherapy plus PORT, followed by chemotherapy alone, PORT alone and observation (5-years OS: 47.4%, 34%, 21.3%, 16.6%, respectively) [7]. Although retrospectively derived on a relatively small sample size, these results provide intriguing data on the effect of modern PORT after optimal adjuvant chemotherapy.

Data from more recent series (although retrospective or community-based) showed a decreasing treatment related death rate with modern techniques such as 3-dimensional (3D) or imaging guided (IMRT) to minimize irradiation of normal tissues (heart and lungs) and maximize the optimal delivery to the targeted fields [42]. A better selection of patients (i.e. only those with extended mediastinal involvement [43] or at higher risk of relapse [44]) may potentially increase the PORT therapeutic index.

Although large, well-designed, prospectively trials evaluating the efficacy of modern PORT are required, the CALGB 9734 prematurely closed due to slow accrual. The Lung Adjuvant Radiotherapy Trial (Lung ART-NCT00410383) comparing 3D-conformal PORT with no PORT in resected N2 patients after the delivery of any planned (neo)-adjuvant chemotherapy is currently ongoing.

## Treatment efficacy according to age

Older age and comorbidities may profoundly affect treatment tolerability and overall mortality rate. Few trials have been specifically conducted in elderly (and frail) patients; thus, the vast majority of data derive from retrospective analyses of randomized clinical trials designed for an adult population.

In the subgroup analysis from the JBR-10, no differential effect favoring adjuvant chemotherapy according to age (cut-off 65-years) was found; indeed, in the 155 patients over 65-s, the HR for death still favored adjuvant treatment (0.61; 95% CI 0.38-0.98; p = .04), in spite of the smaller cumulative doses of cisplatin and vinorelbine [45].

The update of the LCCG meta-analysis did not show differential effect of adjuvant chemotherapy according to age [23], as well as the LACE pooled analysis. In addition, no difference in severe toxicity were encountered according to age (lower cumulative doses?)[46].

A recently published practice-based survey from SEER registry showed that platinum based ACT administered outside of clinical trials to unselected elderly patients was associated with a significant survival benefit (although limited to those under 80-years and associated with a higher risk of serious adverse events)[28]. Moreover, the data from the Cancer Ontario Registry confirmed, once again, the benefit of ACT in the elderly populations (even if any apparent increased toxicity was observed)[26].

The LCCG meta-analysis did show a significant trend (p = 0.002) against the adoption of adjuvant chemotherapy in patients with a worsening performance status (PS) [23] In this regard, LACE subgroup analysis showed increasing benefits from adjuvant chemotherapy with better PS (0 vs 1), with a detrimental effect for PS 2 (test for trend p = .009 for OS) [18].

These results suggest no differential effect of adjuvant chemotherapy when age is the only variable [47], at least when interpreting data from the available randomized clinical trials; whether the daily elderly patient might derive the same benefit from adjuvant chemotherapy should be weighted in the context of comorbidities and other prognostic factors.

## Cisplatinum-based chemotherapy: is there a 'winning' doublet?

Clear and definitive evidence with regard to which would be the best partner to be associated with adjuvant cisplatinum is still awaited. The current opinion, generally shared by ASCO, NCCN, ACCP, and ESMO, is that any cisplatinum-combination (according to the approved dose and schedules for advanced setting) may be administered to patients who have undergone radical resection and who are (at least apparently) disease-free after surgery [1-5]. In addition, doses and schedules should be tailored according to the patients' compliance and the physicians' attitude ("practitioners adopt one cisplatin-based chemotherapy regimen to use consistently to ensure familiarity and optimize patient safety") [2]. This 'opened-minded' instead of 'rigorous' interpretation of available scientific evidence represents a matter of discussion, although it should be recognized that clear recommendations with modern regimens for the daily practice are lacking and are still far to be produced.

With regard to the available evidences to date, the combination cisplatinum plus vinorelbine should be considered to have a 'groundless supremacy'. Indeed, in the prospectively planned subgroup analysis from LACE, cisplatinum and vinorelbine trended toward a major benefit (HR = 0.80; 95% CI. = 0.7-0.91; p < .001) if compared to other regimens (interaction test p = .004) [18], and this benefit was stage dependent (interaction p = .02).

Currently, two issues should be considered: a) patients receiving > 300 mg/m2 of cisplatinum performed better than those receiving < 300 mg/m2. This featured patient subgroup overlaps for almost 65% with that of patients receiving vinorelbine, in comparison with half of those receiving other regimens. Whether the benefit of cisplatinum and vinorelbine depends on the combination of the 2 drugs or from higher cisplatinum dose cannot be easy established [18]; b) the planned schedule of cisplatinum was 50 mg/m2 day 1 and 8 in JBR10 and 100 mg/m2 day 1 in ANITA; vinorelbine in both trials was meant to be delivered 25-30 mg/m2 weekly for 4 cycles (16 doses). In JBR10 the actual dose was 84% and 52% for cisplatinum and vinorelbine respectively, with higher toxicities occurring in older, female and after pneumonectomy [48]. In ANITA only 50% of patients completed the planned 4 cycles.

## The 'fading effect' of chemotherapy

According to the breast or colon cancer models, the benefit of adjuvant treatment may vary over time; the data from NSCLC are conflicting. Long term effect of platinum based ACT was maintained in ANITA after 5 years and in the 7-years (projected) analysis (OS benefit of 8.6% and 8.4%, respectively)[7] and in JBR10 (absolute OS benefit of 11%, after 9.3 years and 12% at 5 years)[9]. However the updated results of CALBG 9633 [13] and IALT [11] did rise many concerns.

CALBG 9633 first analysis (at 2.8 years) showed a promising 11% OS increase in stage IB, which lead to early stopping of the study [12]. Unfortunately this was no longer confirmed after the 4.5 [49] and 6 years updates [13]. In the IALT trial (the largest with 1867 patients), the OS benefit after the 90 months analysis was less evident (and not statistically significant anymore) in comparison with the analysis performed at 56 months (HR 0.91 and 0.56, respectively). The rate of non-lung cancer related deaths increased by 20%, as compared with the first interim analysis, mostly after 5 years of follow up [11]. Although the unbalanced population taken into account after the 5-years time-point should to be considered as a randomized comparison, long term side effects of citotoxic drugs and the high rate of comorbidities in NSCLC patients may partially explain these results [50]. However some differences in classification and reporting of death causes may have influenced the reported outcomes [17].

LACE data show a sustained effect of ACT over time (survival gain of 3,9% and 5,4% at 3 and 5 years, respectively). Considering only lung cancer-related deaths, the benefit was even higher (+ 6,9% at 5 years), partially outweighed by the higher rate of non lung cancer-related deaths observed in the ACT group.

## The integration of bio-molecular predictors in the risk assessment process: are they ready for prime time?

An effective risk assessment is essential to identify "high-risk" stage IB (IA?) patients benefiting from ACT and spare some "low-risk" stage II from the toxicities of a treatment not impacting on their OS. Which factors should be considered in this clinical decision process?

### Clinico-pathological factors

Pathological stage is the only prospectively validated prognostic factor to guide the prescription of adjuvant chemotherapy, although based on inadequate prognostic power to stratify patients within the same TNM category [51,52]. Older age, male gender, poorer PS and non-squamous cell histology are currently known to be associated with decreased survival, although their additional weight to clinical staging does not increase its prognostic power [53].

We previously investigated the independent prognostic role of the number of resected nodes and of the ratio between metastatic and resected nodes. The generated prognostic model was able to powerfully stratify patients into 3 classes [54]. Recently, a large retrospective analysis from the SEER database showed that the increasing number of resected positive nodes and a higher ratio between metastatic and overall resected nodes have an independent negative prognostic impact for overall survival in N1 patients [55,56]. Although a prospective validation is mandatory, these results suggest the inclusion in the next TNM of other nodal descriptors than site and status to improve the prognostic power.

### Molecular factors

Several molecular prognostic (and predictive) models have been published in the attempt to improve the clinical decision process [57-62]. Although promising, their effectiveness and clinical utility were undermined by several limitations: the inability to account for comorbidities and other clinical factors (which affect prognosis) [63], methodological and statistical biases, lack of a solid and reproducible internal and external validation [64,65].

The proposal of a new prognostic model should always be supported by reliable validations. A pivotal example is represented by the recent retraction by Potti et al of their promising metagene expression model (which led to stop the CALBG 30506 trial and to remove the metagene analysis from the study design) [66].

The analysis of the data from the available randomized trials exploring the role of eventual predictors for either prognosis or treatment efficacy hides many drawbacks given its retrospective nature. This is further complicated by the extremely relevant impact of the attrition rate for the analysis of biological samples [67]. Nevertheless, many investigators involved in those trials exploring the benefit of adjuvant chemotherapy planned and conducted intriguing analyses to generate working hypotheses for future biomarker-driven randomized trials, as follows:

• *IALT-BIO: *the low IHC expression of the excision repair cross complementation group 1 (ERCC1) represents a marker of better outcome in patients receiving cisplatinum in ACT (HR = 0.65; p = .0002 vs HR = 1.14; p = .4 for high expression; interaction test p = .0009); conversely, high ERCC1 expression correlates with longer OS in the control group (HR = 0.66) [68]. In the context of cell cycle regulators, p27, while having a predictive role for patients treated with ACT, does not affect prognosis (p27 negative HR 0.66; p = .006; p27 positive HR 1.09; p = .54; interaction test p = .02)[69]. Similarly to ERCC1, the low expression of MutS homologue 2 (MSH2) was predictive of benefit from platinum based -ACT (low MSH2 HR = 0.76; p = .03 vs high MSH 2 HR = 1.12; p = .48; interaction test p = .06). MSH2 high expression was also prognostic for longer survival in untreated patients (HR = 0.66; p = .01)[70]. In order to test the prognostic power of the combination of these 3 markers, patients were clustered into 4 categories according to low/high level of MSH2/ERCC1: the benefit from ACT decreased with the increasing number of positive markers (HR for low ERCC/low MSH 20.65; p = .01). Similar results were shown for p27/ERCC1. Nevertheless, the prognostic effect decreased over time [70]. The other analyzed markers had a weaker or null predictive role [71,72].

• *JBR-10-[BIO]*: K-RAS wt and p-53 wt patients seemed to benefit more from ACT with cisplatinum and vinorelbine (vs mutants) although the interaction test for treatment effect was not significant. P53 expression was prognostic of worse OS in the control arm (HR = 1.89; p = .03), while in the treatment arm it had a positive predictive role (HR = 0.54; p = .02)[73]. From JBR-10 dataset an m-RNA based-15 gene signature was proposed to differentiate high from low risk patients. The HR for death in the observation group was 18 (adjusted at multivariate analysis; 95% CI 5.12-44.04; p < .001). The prognostic power was validated on 4 separate dataset and by RT-PCR on the original dataset. The positive predictive role was confirmed for high risk group (HR of death 0.33; 95% CI 0.17-0.63; p = .0005) but not for low risk (HR = 3.67; p = .21). The external, prospective validation is awaited to confirm these results [74]. Although unpowered to assess the prognostic or predictive impact of EGFR mutation and copy number, a possible trend toward a positive predictive role of the mutation (and copy number) was proposed in JBR10.

• *LACE BIO (ANITA, JBR10, IALT and CALBG 9633): *High class III beta tubulin (TUBB3) expression maintained the negative prognostic impact seen in previous analysis (HR for death = 1.3; p = .001). In metastatic setting, high TUBB3 expression caused resistance to tubulin-targeting agents [75]. No effect in adjuvant setting was detected (interaction test p = .20), but only a trend toward a major benefit for high expression [76]. Other analyses were performed to assess the prognostic and predictive value of p-53 and KRAS. While neither P53 IHC expression nor mutation were prognostic for survival, a trend toward a positive predictive role was seen in wild type patients (significant for squamous cell) [77]. Regarding KRAS, a non significant trend toward a worse OS was seen for mutated patients (significant only for non squamous non adenocarcinoma), with predictive role [78].

• *Other studies: *additional potential biomarkers or classifiers involving different pathways (DNA methylation, mTOR, cytoskeleton protein expression) have been retrospectively evaluated in other studies. Results are promising but should be validated in prospective larger randomized clinical trials [79-82].

## The target therapy paradox

The biomarker-selection approach, i.e. the treatment assignment according to the expression of featured molecular/classifier signatures (for example ERCC1 and BRCA1 for cisplatinum, RRM for gemcitabine) is the basis of many ongoing clinical trials in order to further optimize and customize ACT (table 1).

**Table 1:** Ongoing clinical trials in adjuvant setting

Trial/Acronym	Stage	Marker	Design	Notes
RADIANT	I-IIIA	EGFR IHC pos	4 cycles of optional ACT followed by 2 years of TKI or placebo	• Phase III, placebo controlled; Erlotinib 150 mg/die × 2 years • Primary endpoint: DFS

ECOG 1505	IB (> 4 cm)-IIIA	None	Clinician choice ACT vs Clinician choice ACT + Bevacizumab	• Phase III (2:1); Bevacizumab 15 mg/kg/3 wks • Primary endpoint: OS • Secondary endpoint: DFS, toxicities

SCAT	I-IIIA	BRCA	CDDP DOC vs "gen assigned CT"	• Phase III (3:1) CDDP DOC/DOC/CDDP GEM vs CDDP/DOC • Primary endpoint: DFS • Secondary endpoint: OS, toxicities

TASTE	I-IIIA	ERCC1/EGFR mutant	CDDP/ERL	• Phase II feasibility

SWOG 0720	I	ERCC1/RRM1	Follow up or ACT according to risk profile	• Phase II feasibility

MAGRIT	IB-IIIA	MAGE A3 +	Vaccine vs placebo after optional ACT	• Phase III (2:1) placebo controlled; separate analysis for ACT +/- • Primary endpoint: DFS • Secondary endpoint: OS

ITACA	II-IIIA	ERCC1/TS	ACT assigned according to markers expression	• Phase III • Primary end-point: OS

TREAT	IB-IIIA	None	CDDP PEM vs CDDP GEM	• Randomized phase II • Primary endpoint: feasibility • Secondary endpoint: toxicity, delivery

EGFR: epidermal growth factor receptor; IHC: immunohistochemistry; ACT: adjuvant chemotherapy; TKI: tyrosine kinase inhibitors; DFS: disease-free survival; OS: overall survival; CDDP: cisplatin; DOC: docetaxel; GEM: gemcitabine; ERCC: Excision Repair Cross-Complementation gene; RRM: ribonucleotide reductase M1 gene; MAGE: melanoma-associated antigen; TS: thymidylate synthetase; PEM: pemetrexed.

While target therapy has dramatically changed the treatment of advanced stage NSCLC, its results in resected NSCLC are currently disappointing. Indeed, the JBR-19 trial, designed to evaluate the benefit of Gefitinib regardless of chemotherapy in unselected patients with resected stage IB-IIIA, did not show any benefit over placebo (and prematurely closed on the basis of the negative results of the ISEL [83] and SWOG 0023 trials [84]) [85]. Moreover, although underpowered to draw definitive conclusions (500 patients out of the 1200 planned), even in the EGFR-mutant patient subgroup no difference in favor of Gefitinib was determined. In this trial, both EGFR mutation/amplification and K-RAS status were neither prognostic for OS nor predictive of outcome.

These disappointing data suggest that: 1) the efficacy of the administration of a targeted agents in patients expressing a sensitizing biomarker, may be diluted in the context of an unselected population; 2) the biological background behind advanced and surgically removed disease can be extremely different. In this direction, retrospective analyses, such as the one from the MSKCC, indicate a potential benefit (a trend toward better disease-free survival) of Gefitinib or Erlotinib after adjuvant chemotherapy in EGFR-mutant patients [86]. The results of the ongoing RADIANT trial, randomizing only EGFR positive patients (IHC or FISH) to 2 years of Erlotinib vs placebo after optional ACT, are awaited.

## Conclusions

ACT for radically resected NSCLC is now part of the routine clinical approach to early NSCLC and is certainly contributing to the decrease in mortality observed in these patients in recent years. While many important 'technical' questions, such as optimal treatment for Stage I patients, best platinum based combination, and optimal use of PORT to name a few, remain to be answered to further refine currently achievable results, the biggest challenge ahead is to better understand the underlying biology of the disease and to incorporate biological advances into clinical treatment algorithms. Ongoing adjuvant trials, such as the italian ITACA, will hopefully assess the role of pharmacogenomically 'tailored' ACT to optimize the use of currently available classical cytotoxic agents; however, genetic and epigenetic drivers of early NSCLC must be clearly identified in order to generate a further 'leap' in the management of resectable NSCLC patients, both in terms of accurate prognostication and risk assessment and in terms of better prediction of sensitivity/resistance to specific targeted treatments. The ever growing knowledge on molecular pathways, cancer stem cell populations, and genetic/epigenetic programs regulating the invasive and metastatic phenotype will shed new light on the right path to be undertaken in order to ensure the best treatment to each specific patient population.

## Competing interests

The authors declare that they have no competing interests.

## Authors' contributions

All named authors conceived for the study, participated in its design and coordination and helped to draft the manuscript. All authors read and approved the final manuscript.
